# Controllable Molecule Transport and Release by a Restorable Surface-tethered DNA nanodevice

**DOI:** 10.1038/srep28292

**Published:** 2016-07-07

**Authors:** Zhaoyin Wang, Yuanyuan Xu, Haiyan Wang, Fengzhen Liu, Zhenning Ren, Zhaoxia Wang

**Affiliations:** 1 State Key Laboratory of Pharmaceutical Biotechnology, Department of Biochemistry, Nanjing University, Nanjing, 210093, P R China; 2 Key Laboratory of Animal Physiology and Biochemistry, College of Veterinary Medicine, Nanjing Agricultural University, Nanjing, 210095, China; 3 Department of Oncology, the Second Affiliated Hospital of Nanjing Medical University, Nanjing, 210011, China

## Abstract

In this paper, we report a novel surface-tethered DNA nanodevice that may present three states and undergo conformational changes under the operation of pH. Besides, convenient regulation on the electrode surface renders the construction and operation of this DNA nanodevice restorable. To make full use of this DNA nanodevice, ferrocene (Fc) has been further employed for the fabrication of the molecular device. On one hand, the state switches of the DNA nanodevice can be characterized conveniently and reliably by the obtained electrochemical signals from Fc. On the other hand, β-cyclodextrin-ferrocene (β-CD-Fc) host-guest system can be introduced by Fc, which functionalizes this molecular device. Based on different electrochemical behaviors of β-CD under different states, this DNA nanodevice can actualize directional loading, transporting and unloading of β-CD in nanoscale. Therefore, this DNA nanodevice bares promising applications in controllable molecular transport and release, which are of great value to molecular device design.

In the preceding decades, many molecule devices with excellent structures and functions were fabricated^1^,^2^,^3^,^4^. Among the various materials utilized in molecule device construction, DNA is an ideal building block for bottom-up design because of its unique properties in base pairing and structural diversity^5^,^6^. Besides the simple structure such as double helix^7^, DNA can also form, under certain modulations, complex structures, including i-motif 8,9, G-quadruplex^10^,^11^, triplex structure^12^,^13^,^14^. All merits of all these structures may facilitate the design of DNA nanodevices, making DNA nanodevices the most promising ones.

Currently, the colleagues have made great progress in DNA nanodevice construction. To be noted, Fan and coworkers recently reported a series of pioneering work on three-dimensional (3D) DNA tetrahedral nanodevice. It is demonstrated that the configuration of the surface-confined 3D DNA tetrahedral nanodevice could be switched via the stimulation of DNA^15^. Meanwhile, 3D DNA tetrahedral nanodevice tethered on the interface is highly uniformed and exhibit unique advantage in bioanalytical methods over the double stranded DNA^16^,^17^,^18^. More importantly, relying on the precise size of DNA, 3D DNA tetrahedral nanodevice could be constructed with controllable size, showing great potential in various biosensors^19^,^20^,^21^. Besides, since aptamer, a type of special DNA, possesses biorecognition function, the importance of aptamer in DNA nanodevice has also been surveyed^22^. However, significant challenges, especially in the demand for functionality and restorability of the DNA nanodevices, still remain. Meanwhile, functional DNA nanodevices still lack in variety, although some ingenious DNA nanodevices have been reported for the applications to molecular detection^23^, multistep synthesis^24^, programmable assembly^25^ and protein binding affinity regulation^26^. It is also worthy of attention that the useful DNA nanodevices are usually much more complex than the prototype DNA nanodevices. Actually, it is the structural complexity that ensures these DNA nanodevices working in functional ways. Hence, in order to design more useful DNA nanodevices, diverse DNA structural regions should be integrated into one DNA nanodevice. One step further, these integrated structural domains should be well-organized and coordinated, so that they serve as a whole entity and would not interfere with each other. Moreover, the functionality of the devices may take another great leap if these devices are reversible and repairable molecular gadgets rather than mere disposable products.

In this study, we have devised a novel DNA nanodevice consisting of three components. Working concertedly, these components may present three states, “duplex state”, “single state” and “i-motif-triplex state”, upon the stimulation of pH. What is more, since the DNA nanodevice is constructed on an electrode surface, reversibility and reparability of this device can be easily achieved and each step of the DNA nanodevice operation can be observed via the obtained electrochemical signals. Furthermore, to make the application of this surface-tethered DNA nanodevice explicit, “β-cyclodextrin-ferrocene” (β-CD-Fc) host-guest system has been introduced in this nanodevice. While, Fc, which is attacked at one end of DNA, is used for the electrochemical signal readout as the redox probe^27^,^28^,^29^, as a hydrophobic group, it can also form an inclusion complex with β-CD which has a hydrophobic cavity^30^,^31^,^32^. More interestingly, since Fc can be changed into the electron-deficient form of Fc^+^ upon electrochemical oxidization, Fc^+^ can be released from the β-CD-Fc inclusion complex because the positive charge may greatly impair its hydrophobicity^33^,^34^. Therefore, a novel DNA nanodevice has been built, and this device can present actual function of loading, transporting and unloading molecules under the control of pH and electrochemical treatment.

## Results and Discussion

### Construction and operation of the surface-tethered DNA nanodevice

**Figure 1:**
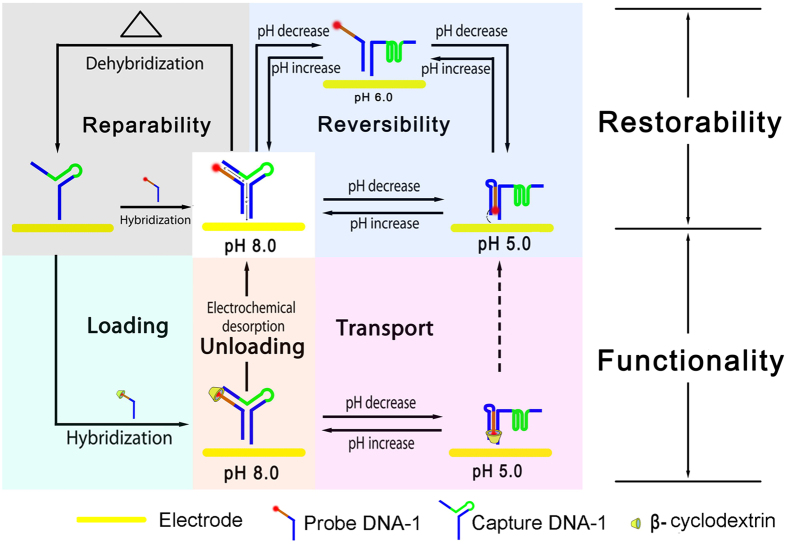
Schematic illustration of the DNA nanodevice construction and operation with the characters of functionality and restorability.

**Figure 2:**
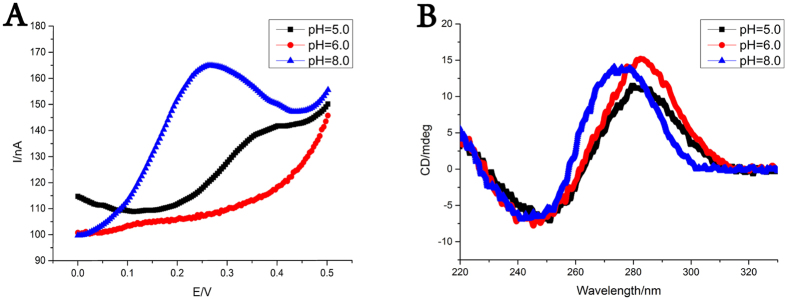
(**A**) DPV curves for different electrochemical signals of the DNA nanodevice at different pH. (**B**) Circular dichroism spectra of the DNA nanodevice in PBS buffers with different pH.

Two DNA single strands, i.e., a thiol modified Capture DNA-1 and an Fc modified Probe DNA-1, are employed for the construction of the nanodevice. Capture DNA-1 contains an i-motif sequence and can form a hairpin structure. Probe DNA-1 rich in cytosine is designed to hybridize with the single strand section of the hairpin Capture DNA-1. These two strands can be combined into a DNA nanodevice that may present three state switches under the operation of pH in details. The procedures of DNA nanodevice construction and operation are as follows (Fig. 1). Firstly, Capture DNA-1 is immobilized on an electrode surface by the covalent Au-S bond. Probe DNA-1 is then assembled onto the electrode surface by hybridization and the DNA nanodevice is constructed. In alkaline environment, the DNA nanodevice would form a conformation that resembles as the letter “Y”. This state is defined as “duplex state”. To be noted, the i-motif sequence and triplex sequence have been integrated into the device and would perform their own tasks under the operation of pH. With the decrease of pH, Capture DNA-1 tends to form i-motif structure because the contained i-motif sequence. In this situation, part of the i-motif-related duplex DNA is separated and the sequence flanking Fc is released as a single strand section. This state is defined as “single state”. With further decrease of pH, the single strand section of Probe DNA-1 would form triplex structure due to the protonation of cytosine residues in Probe DNA-1. This state is defined as “i-motif-triplex state”. Upon the switch of the device from “duplex state” to “i-motif-triplex state”, the Fc in the nanodevice is driven close to the electrode surface. It has been known that Fc will present different electrochemical signals with the switches of DNA conformation^35^,^36^,^37^. So, the current value and potential of the peak can reflect the efficiency of electron transfer, which is related to the route and distance of electron transfer^38^,^39^,^40^,^41^. We have then exploited these principles to confirm the operation of the DNA nanodevice exactly. As depicted in Fig. 2A, the signal of Fc changes at pH 8.0, 6.0 and 5.0. The largest peak obtained at pH 8.0 indicates that “duplex state” has been constructed on the electrode surface because only along the duplex strand of DNA can electron transfer so effectively^42^. Similarly, since electron cannot transfer along single strand, nearly no peak can be detected at pH 6.0, which proves that the DNA nanodevice is in “single state” at these pH conditions^43^. According to the fact that the value of peak current becomes larger at more acidic pH, it can be concluded that Fc is driven nearer to the electrode surface thanks to the formation of triplex structure^44^. In this structure, electron can transfer again but in an inefficient way. The conformational changes of this DNA nanodevice have also been confirmed by the results of circular dichroism (Fig. 2B). The circular dichroism spectrum shows a large positive band at about 270 nm, which reflects the formation of B-form duplex DNA. With the decrease of pH, the positive band shifts to higher wavelength with a larger peak value, which reveals the appearance of i-motif structure in our DNA nanodevice. At pH 5.0, the positive band decreases, because two kinds of structures (i-motif structure and triplex structure) exist simultaneously and triplex strand has a typical negative band around 280 nm^45^. The concerted manner in which pH change may act on this DNA nanodevice has riveted our attention. As demonstrated in Figures S1 and S2, with the decrease of pH, i-motif structure and triplex structure tend to form sequentially. I-motif structure forms at higher pH value and releases a single strand, which assists the triplex structure formation at lower pH. It is just like that we equip the device with two engines, which need the same fuel and can work together. Such concerted and coordinated structural changes are different from the previously reported devices and will certainly make our device more powerful.

### Reversibility and reparability of this DNA nanodevice

**Figure 3:**
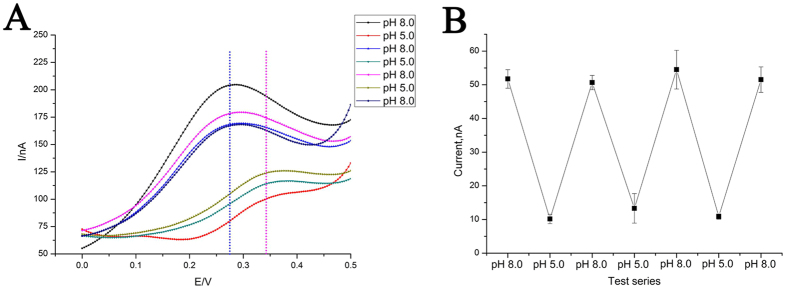
(**A**) Repeated switches between “duplex state” and “i-motif-triplex state” of the DNA nanodevice after sequential operation of pH. Blue dotted line shows the DPV peak potential of Fc at the pH 8.0; purple dotted line shows the DPV peak potential of Fc at the pH 5.0. (**B**) DPV peak current derived from Fig. 2A. The error bars represent the standard deviations of three parallel tests.

After optimizing the concentration of Probe strand-1 (Figure S3), our DNA nanodevice was operated at pH 5.0 and 8.0 for more than seven times. As shown in Fig. 3, we can still obtain the typical signals of Fc in “duplex state” and “i-motif-triplex state”. Such repeatable signals have manifested the reversibility of our DNA nanodevice which can be attributed to the two features of our design. One is the choice of H^+^, avoiding the participation of any enzyme in our system which is prone to break the DNA strands. The other is the benign condition of the operation (pH 5.0 or 8.0), which is prone to maintain the integrality of DNA strands. These two factors, combined together, have assured that pH operation of our device would only induce structural switches between “duplex state” and “i-motif-triplex state” instead of any unexpected damage to the component strands of the DNA nanodevice. Therefore, the reversibility of this device can be realized, so our DNA nanodevice is capable of working numerous times after one construction. Compared with the DNA nanodevices fabricated in solution, DNA nanodevices constructed on a solid surface might have more intricate functions, because the conditions used to manipulate the nanodevices can be applied and altered conveniently.

**Figure 4:**
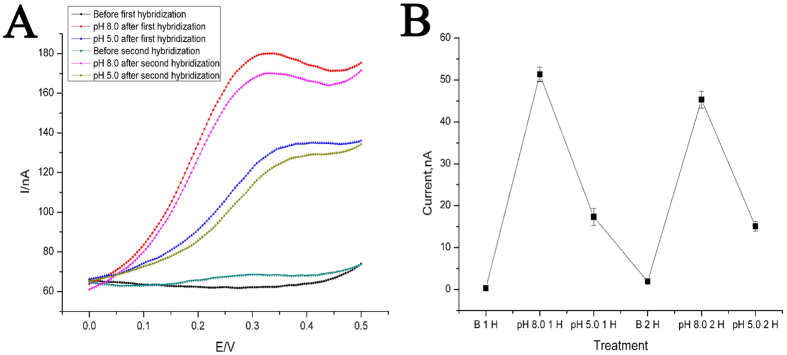
(**A**) DPV curves for the operation of the DNA nanodevice after hybridization and re-hybridization. (**B**) DPV peak current derived from Fig. 3A. Others same as that in Fig. 2.

The other identified character of this DNA nanodevice is reparability. It has been well known that duplex DNA melts into single DNA at high temperature, however, which will not affect the Au-S bond. More interestingly, if the dehybridization occurs on a solid surface, it will be easy to separate Probe DNA-1 from Capture DNA-1 immobilized on the solid surface. So, after the well-constructed electrode is kept in deionized water at 90 °C for 10 min, Probe DNA-1 is released from the electrode surface and DNA nanodevice collapsed into a single Capture DNA-1. Owing to the release of Probe DNA-1, electrochemical signal of Fc cannot be detected. Just like being freshly immobilized onto the electrode, the heat-treated Capture DNA-1 is single again and ready to hybridize with a new Probe DNA-1. In this case, the device can be reconstructed, as evidenced by the re-appearance of Fc signal after re-hybridization. What is more exciting, the DNA nanodevice can be recovered to have the ability of state switching under the operation of pH (Fig. 4), which means that the DNA nanodevice can be repaired simply by heating and re-hybridization. To the best of our knowledge, this DNA nanodevice is the first reparable one and this character sets the basis for repetitive usage of the DNA nanodevices like a real machine. Generally, this restorable DNA nanodevice can work repetitively and is easy to be repaired.

### Controllable molecular transport and release of this DNA nanodevice

**Figure 5:**
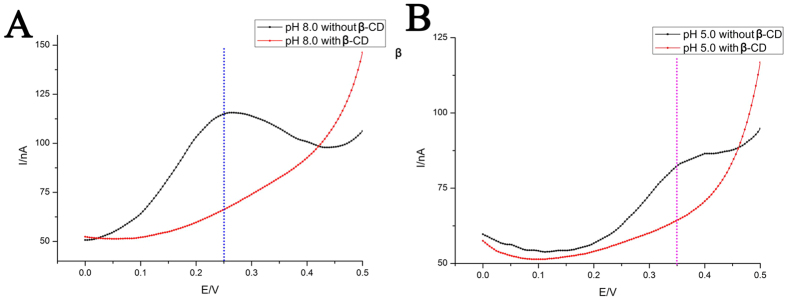
(**A**) DPV curves for “duplex state” of the nanodevice with and without β-CD. (**B**) DPV curves for “i-motif-triplex state” of the nanodevice with and without β-CD. Others same as that in Fig. 2.

We further use our DNA nanodevice in controlled molecular transport and release by employing “β-cyclodextrin-ferrocene” (β-CD-Fc) host-guest system. Since the guest molecule Fc has been modified to the DNA strand, the host molecule β-CD is then loaded, transported and unloaded by this device (Figure S4). The loading process is achieved through two steps. Firstly, β-CD is incubated with the Fc modified Probe DNA-1, thus β-CD-Fc-DNA is formed by hydrophobic interaction between Fc and β-CD^32^. Then, β-CD-Fc-DNA hybridizes with the Capture DNA-1 that is previously immobilized on the electrode surface. Results of the loading process have been characterized (Fig. 5). Compared with the device fabricated with Fc-DNA, the Fc signal of the device fabricated with β-CD-Fc-DNA cannot be detected either in “duplex state” or in “i-motif-triplex state”. This is also consistent with the fact that the signal of Fc has been blocked by the β-CD-Fc inclusion complex^46^,^47^. Therefore, it can be concluded that the “target molecule” β-CD can be loaded onto this DNA nanodevice accompanied with the hybridization of β-CD-Fc-DNA and Capture DNA-1.

**Figure 6:**
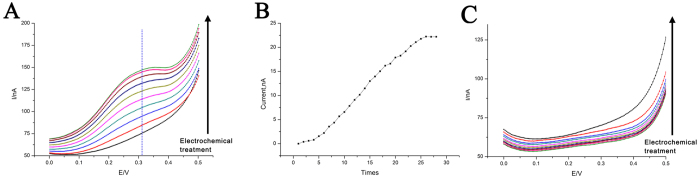
(**A**) DPV curves for electrochemical oxidation of the β-CD loaded DNA nanodevice in “duplex state” for 28 times. (**B**) DPV peak current derived from Fig. 5A. (**C**) DPV curves for electrochemical oxidation of the β-CD loaded DNA nanodevice in “i-motif-triplex state” for 10 times. Others same as that in Fig. 2.

After demonstration of stability of DNA nanomachine (Figures S5 and S6), it is found that release of the β-CD can be realized by electrochemical desorption under certain pH condition. It has been known that the hydrophobic interaction between Fc and β-CD will be diminished upon the oxidation of Fc^33^,^34^. An electrochemical treatment can easily trigger the switches between reduced form Fc and oxidized form Fc^+^ and achieve electrochemical desorption of β-CD from β-CD-Fc inclusion. The effect of electrochemical desorption is related to the efficiency of electron transfer. Since the two typical working states, “duplex state” and “i-motif-triplex state”, display different electron transfer efficiency, β-CD should be released in different degrees when the device is in these different working states. Figure 6 indicates the distinct electrochemical desorption behaviors. When the device is at “duplex state”, the signal of Fc gradually increases with repeated electrochemical treatment. This reflects that some Fc is oxidized to Fc^+^, expelling β-CD from β-CD-Fc inclusion complex. After cycles of electrochemical desorption, more and more Fc can be detected, thus increased signal of Fc can be obtained (Fig. 6A,B). So we can conclude that β-CD can be successfully released in “duplex state” of the DNA nanodevice. Desirably, the DNA nanodevice can show a different behavior in “i-motif-triplex state”. The foregoing experimental results have shown that, in this structure, electron can hardly transfer from Fc to electrode, which determines that it is very difficult to oxidize Fc in this state. As expected, after quite a lot of cycles of electrochemical treatment, nearly no signal can be obtained (Fig. 6C). Based on the different behaviors in different device states, β-CD is released successfully under the operation of dual stimulus, which means suitable pH will provide structural base and electrochemical treatment will trigger the release.

**Figure 7:**
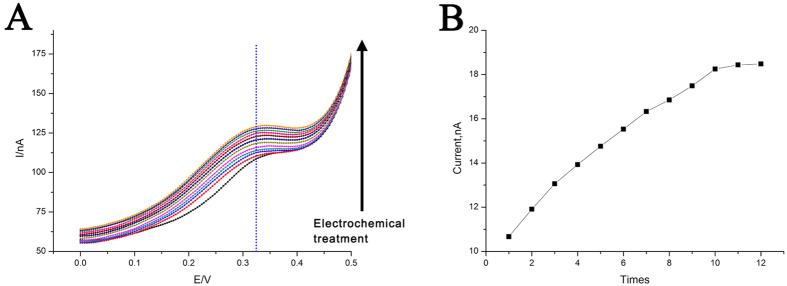
(**A**) DPV curves for electrochemical oxidation of the β-CD loaded DNA nanodevice in “duplex state” for 12 times after the device had been electrochemical treatment for more than 10 times in “i-motif-triplex state”. (**B**) DPV peak current derived from Fig. 6A. Others same as that in Fig. 2.

We have further corroborated that our DNA nanodevice can directionally transport β-CD. With the increase of pH, structure of the DNA nanodevice is switched from “i-motif-triplex state” to “duplex state”. And the distance between β-CD-Fc inclusion complex and electrode surface becomes larger accompanied by this structural switch. Therefore, β-CD is transported and can be released naturally in “duplex state” (Fig. 7A). The fact that the DNA nanodevice can recover the ability of unloading β-CD indicates that our DNA nanodevice possesses the function of directionally molecular transport. In addition, apart from in the electrically driven transportation and releasing based on the β-CD-Fc system, our nanomachine may reveal great potential in other aspects. For instance, based on a stronger interaction between β-CD and adamantine^48^,^49^, a material-stimuli releasing nanosystem could be designed. Besides, by modifying hydrophilic or hydrophobic groups at the terminus of DNA oligonucleotides, transition of hydrophobic-hydrophilic on a solid surface can be achieved with the conformation change of this DNA nanodevice, which is quite valuable in fundamental research and practical applications^50^,^51^. In addition, this device can be used to design different kinds of biosensors by integrating other methods, such as single-molecule fluorescence spectroscopy and surface-enhanced Raman scattering^52^,^53^.

In summary, we have fabricated a novel surface-tethered DNA nanodevice, which presents three states and possesses the function of controllable molecular transport and release under the operation of H^+^/OH^−^ and electron. This device can be repaired and regenerated conveniently, so it can work time and again rather than in a disposable manner, which is the first report for such kind of nanodevices. Taking β-CD as target molecule, this DNA nanodevice achieves load, transport and release β-CD under controlled conditions. Considering the broad applications of host-guest system and the merits of this DNA nanodevice, we believe this surface-tethered DNA nanodevice may enrich the diversity and functions of DNA nanodevices and provide broad applications in controllable molecular transport and release.

## Methods

### Materials

Ethylenediaminetetraacetic acid (EDTA), mercaptohexanol (MCH), sodium hyperchlorate (NaClO_4_), and tris(2-carboxyethyl)phosphine hydrochloride (TCEP) were purchased from Sigma. These were used as received. Other chemicals used in this work were of analytical grade and directly used without additional purification. All oligonucleotides were synthesized and purified through HPLC by TaKaRa Inc. (Dalian, China), and the sequences were as follows.

Capture DNA-1 5′-GGAGGAGGACCCTAACCCTAACCCTAACCCGCGTTAGGGTTAGAGGAGGAGG-C6-SH-3′

Capture DNA-2 5′-GGAGGAGGACCCTAACCCTAACCCTAACCCGCGTTAGGGTTAGAGGAGGAGG-3′

Probe DNA-15′-CCTCCTCCTGGTCCTCCTCC-Fc-3′

Probe DNA-25′-CCTCCTCCTGGTCCTCCTCC-3′

The buffers employed in this work were listed. DNA immobilization buffer: 10 mM Tris-HCl, 1 mM EDTA, 0.1 M NaCl, and 10 μM TCEP (pH 7.4). Hybridization buffer: 10 mM Tris-HCl with 1 M NaClO_4_ (pH 7.4). Washing buffer: 10 mM Tris-HCl solution (pH 7.4). Electrolyte buffer: 0.2 M phosphate buffered saline with 0.1 M NaClO_4_ solution (from pH 4.0 to pH 9.0). All solutions were prepared with ultrapure water (18.2 MΩ · cm) from a Milli-Q system (Bedford, MA).

### Gold electrode pretreatment and DNA immobilization

The substrate gold electrode (diameter 3.0 mm) was soaked in piranha solution (H_2_SO_4_: 30% H_2_O_2_ = 3:1) for 5 min to eliminate the adsorbed organic matter, and then rinsed with water. After that, the electrode was abraded with successively finer grades sand papers and then polished to mirror smoothness with alumina powder of various particle sizes (1.0 and 0.3 μm) on microcloth. Finally, it was sonicated for 5 min in both ethanol and water and electrochemically activated in 0.5 M H_2_SO_4_ until a stable cyclic voltammogram was obtained. The electrode with DNA self-assembly monolayers (SAMs) were obtained by incubation with 1.0 μM Capture DNA-1 for 16 h at room temperature, followed by 1 h treatment with an aqueous solution of 1 mM MCH to get well-aligned DNA monolayers. The electrode was then rinsed with pure water, dried again with nitrogen, and stored at 4 °C prior to use.

### The assembly and disassembly of DNA nanodevice

The assembly of the DNA nanodevice was simply accomplished by immersing Capture DNA-1 modified electrode into 1 μM Probe DNA-1 solution for 2 h at 37 °C. Then, the electrode was then rinsed with washing buffer thoroughly for further release procedure. DNA nanodevice disassembly was achieved by immersing the electrode in deionized water for 10 min at 90 °C.

For fabrication of a nanodevice consisted of β-CD, 10 μL Probe DNA-1 was first mixed with 1 μL 100 mM β-CD and 89 μL hybridization buffer in water bath at 37 °C for 2 h. Then, Capture DNA-1 modified electrode was immersed into the above solution at 37 °C for another 2 h.

### The release procedure of the DNA nanodevice

The release procedure was carried out by electrochemical treatment at different pH. Specifically, the states, “duplex state” at pH 8.0 and “i-motif-triplex state” at pH 5, were regulated firstly. Then, at a certain pH, loaded DNA device suffered the scan of voltage from 0.5 V to 0 V. The electrochemical treatment was repeated, and the signal was recorded.

### Circular dichroism spectroscopy

To prepare DNA nanodevice in different pH, unmodified capture DNA and probe DNA (Capture DNA-2 and Probe DNA-2) were firstly dissolved in PBS with different pH and then mixed in equal volume. After heated for 10 min at 90 °C, the mixed solution cooled down slowly to room temperature in 2 hour. Then DNA nanodevice was ready for circular dichroism measurements. CD measurements were carried out on a JASCO (J-810) CD spectrometer using quartz cells and a 2 nm band width, 0.5 s response, 0.1 nm data pitch, and 100 nm · min^−1^ scanning speed. The scan range was from 350 nm to 200 nm. All the experiments were performed at room temperature.

### Electrochemical analysis

Electrochemical measurements were carried out on 660D Electrochemical Analyzers (CH Instruments) with a conventional three-electrode cell at room temperature. The three-electrode system consisted of a saturated calomel electrode (SCE) as the reference electrode, a platinum wire as the counter electrode, and a gold electrode as the working electrode. Differential pulse voltammetry (DPV) experiments were performed in electrolyte buffers with different pH. The parameters of all DPV experiments were set as the potential range from 0.5 V to 0 V with a pulse amplitude of 50 mV and a width of 50 ms.

## Additional Information

**How to cite this article**: Wang, Z. *et al*. Controllable Molecule Transport and Release by a Restorable Surface-tethered DNA nanodevice. *Sci. Rep*. **6**, 28292; doi: 10.1038/srep28292 (2016).

## Supplementary Material

Supplementary Information
