# Supplementary material for: Controllable Molecule Transport and Release by a Restorable Surface-tethered DNA nanodevice
Source: Sci Rep. 2016 Jul 7;6:28292. doi: 10.1038/srep28292 (PMC4935947; doi:10.1038/srep28292)
Supplement: Supplementary Information [file srep28292-s1.pdf]

Electronic Supplementary information
For
Controllable Molecule Transport and Release by a Restorable Surface-tethered DNA nanodevice

Zhaoyin Wang[1,+], Yuanyuan Xu[1,2,+,*], Haiyan Wang[1], Fengzhen Liu[3], Zhenning Ren[1] & Zhaoxia Wang[3,*]

[1] State Key Laboratory of Pharmaceutical Biotechnology, Department of Biochemistry, Nanjing University, Nanjing 210093, P R China

[2] Key Laboratory of Animal Physiology and Biochemistry, College of Veterinary Medicine, Nanjing Agricultural University, Nanjing 210095, China

[3] Department of Oncology, the Second Affiliated Hospital of Nanjing Medical University, Nanjing, 210011, China

---

[*]Corresponding authors. *E-mail addresses*: xuyuanyuan@njau.edu.cn (Y. Xu), zhaoxiawang88@hotmail.com (Z. Wang).

+These authors contributed equally to this work.

SUPPLEMENTAL RESULTS

1. Conformation of the DNA nanodevice with circular dichroism

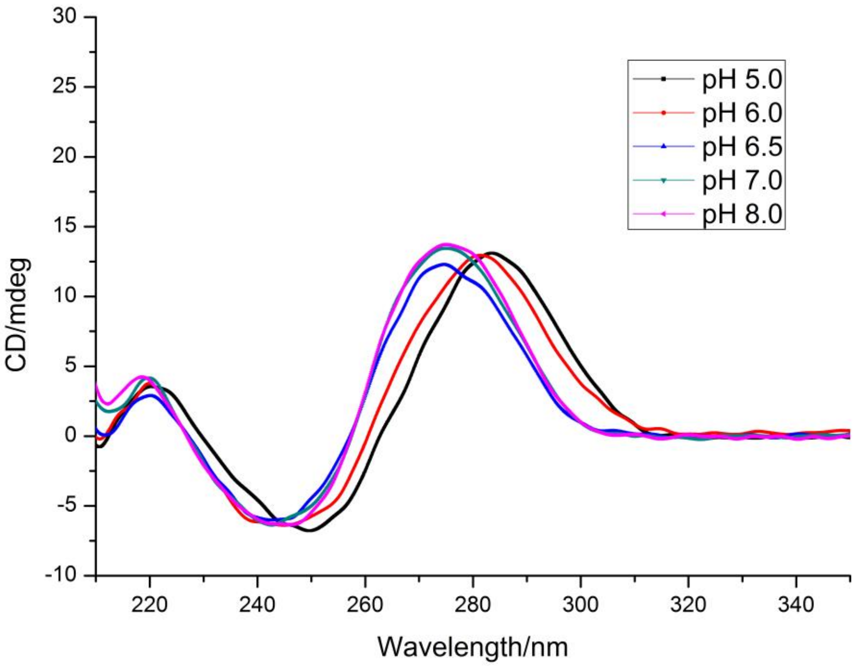

Figure S1 Circular dichroism (CD) Spectra of the DNA nanodevice with different pH.

CD spectroscopy can reflect the structure of DNA strands. Figure S1 depicts the structural changes of our DNA nanodevice caused by pH. CD spectrum showed a large positive band at about 270 nm, which reflected the formation of B-form duplex DNA. With the decrease of pH, the positive band shifted to higher wavelength, which proved the appearance of i-motif in our DNA nanodevice.

2. Confirmation of the DNA nanodevice with differential pulse voltammetry

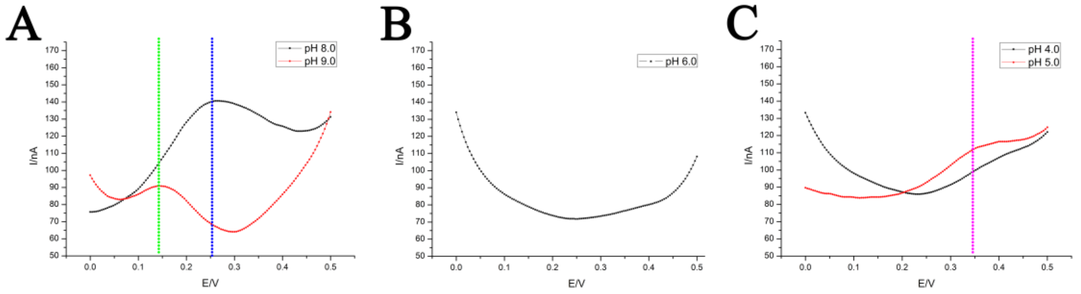

Figure S2 Differential pulse voltammetry (DPV) curves of the DNA nanodevice at different pH.

Ferrocene (Fc) presents different electrochemical signals with the switch of DNA conformation, thus electrochemical experiments were accomplished to confirm the entire process (Figure S2). The largest peak obtained at pH 8.0 indicates that double-stranded structure has been constructed on the electrode surface because only along the duplex strand of DNA can the electron transfer so effectively. Since electron cannot transfer along single strand, nearly no peak can be detected at 6.0. Since the value of peak current becomes larger at more acidic pH, it can be concluded that Fc is driven nearer to the electrode surface thanks to the formation of triplex structure. In other experimental environments, such as higher or lower pH, the stability of DNA structures will be affected. Therefore, the peaks at pH 4.0 or 9.0 are smaller than those at pH 5.0 or 8.0.

3. Effect of probe strand concentration

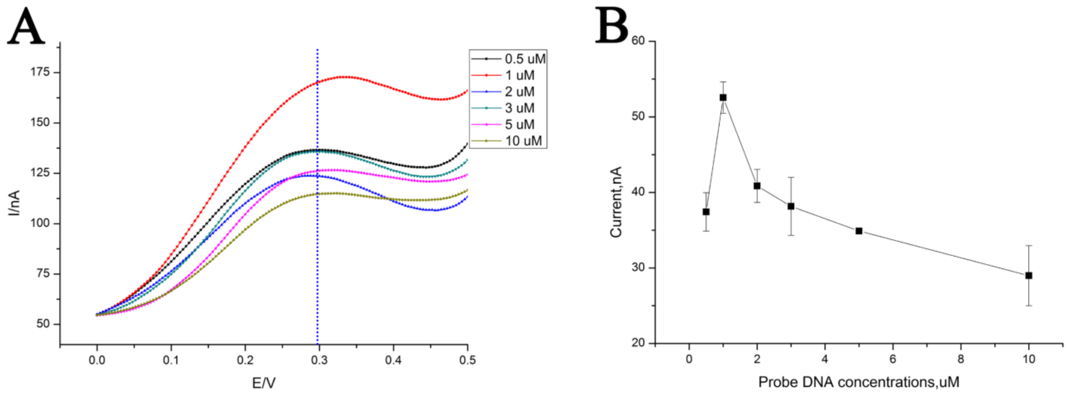

Figure S3 (A) DPV curves for different concentrations of Probe DNA-1 hybridized with 1 μM Capture DNA-1 modified on electrode surface. Blue dotted line shows the DPV peak potential of Fc at the pH 8.0. (B) DPV peak current derived from Figure S1A. The error bars represent the standard deviations of three parallel tests.

Figure S3 depicts the optimization of Probe strand-1 concentration for nanodevice construction. It can be concluded that the DNA nanodevice with probe concentration of 1 μM shows the highest current response. Higher hybridization concentration (>1μM) may probably generate stronger steric hindrance effect of the DNA, while lower concentration of probe strand cannot provide enough hybridization for Capture strand-1. Based on Figure S1, an optimal probe strand concentration of 1 μM was chosen for the construction of our DNA nanodevice.

4. Effect of β-CD concentration

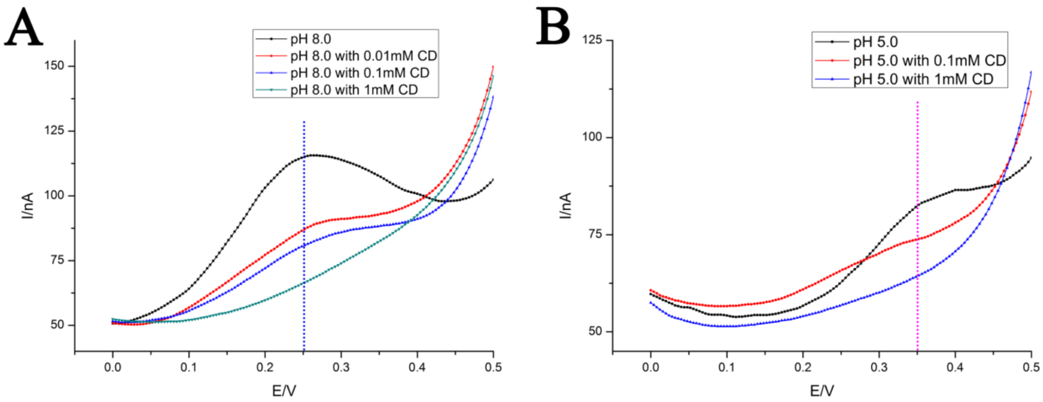

Figure S4 DPV curves for different concentrations of β-cyclodextrin (β-CD) loaded on the DNA nanodevice at (A) pH 8.0 and (B) pH 5.0. Blue dotted line shows the DPV peak potential of Fc at the pH value 8.0; purple dotted line shows the DPV peak potential of Fc at the pH 5.0.

Figure S4 shows the experimental results of β-CD concentration variation at different pH. We noticed that the zero background signals can be achieved when the concentration of β-CD reaches 1 mM at pH 8.0 and 5.0. Therefore, an optimal β-CD concentration of 1 mM was chosen for realizing the loading function of our DNA nanodevice.

5. The stability of this DNA nanodevice without β-CD in aqueous solution

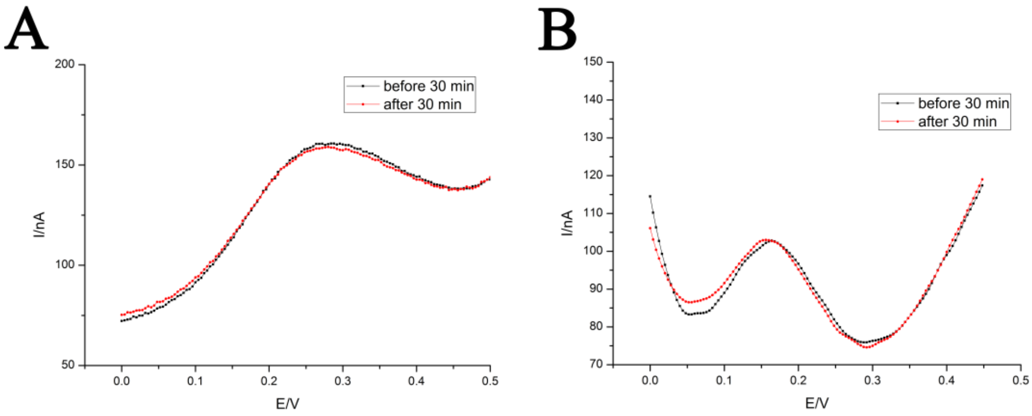

Figure S5 DPV curves for the DNA nanodevice immersed in (A) pH 8.0 or (B) pH 9.0 PBS buffer before and after 30 min.

As shown in Figure S5, the electrochemical signal was nearly unchanged, after the DNA nanodevice was immersed in buffers with pH 8.0 or 9.0 for 30 minutes, which proves the stability of our DNA nanodevice.

6. The stability of this DNA nanodevice with β-CD in aqueous solution

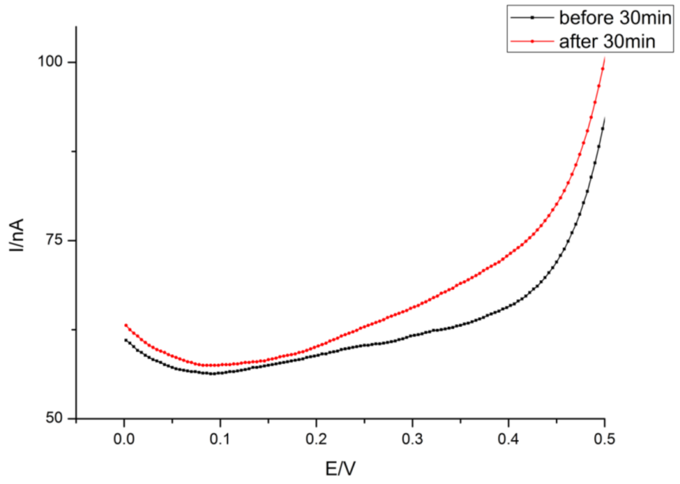

Figure S6 DPV curves for the β-CD loaded DNA nanodevice immersed in pH 8.0 PBS buffer before and after 30 min.

The stability of β-CD incubated with Probe DNA-1 is essential for identifying the unloading function of this DNA nanodevice. As shown in Figure S6, the β-CD loaded nanodevice was not disturbed by 30 min PBS solution soaking, as evidenced by no change in current response. So, it can be confirmed that the unloading of β-CD was not caused by self-desorption.
